# Transcranial Pulse Stimulation (TPS) as a suitable add-on treatment of patients with Alzheimer’s Disease?

**DOI:** 10.1192/j.eurpsy.2026.10663

**Published:** 2026-07-31

**Authors:** V. Rößner-Ruff, C. A. Penkov, J. T. Michaelsen, J. Krieger, K. Friedrich, M. Ziegenbein

**Affiliations:** Research & Development, Wahrendorff Clinic, Sehnde, Germany

## Abstract

**Introduction:**

Methods of neurostimulation have been used to treat neurological and neuropsychiatric disorders. Several methods of non-invasive brain stimulation have been investigated, including electroconvulsive therapy (ECT), transcranial magnetic stimulation (TMS), transcranial direct current stimulation (tDCS) and transcranial pulse stimulation (TPS). While ECT, TMS and tDCS have been studied extensively, there is an urgent need for more research on TPS. In the context of Alzheimer’s disease, for example, some studies have demonstrated favorable outcomes when TPS was used as a neurostimulation method. However, this method has yet to be incorporated into clinical practice due to the inability to conclusively assess its effectiveness, which is influenced by several factors.

**Objectives:**

A new method of neurostimulation, TPS, will be presented. The results of a recent study that used TPS on patients with mild to moderate Alzheimer’s disease (AD) will also be presented. The study aims to investigate whether cognitive performance increases significantly and depressive symptom severity decreases significantly.

**Methods:**

Cognitive performance (MoCA) and depressive symptoms (GDS) of patients with mild or moderate early- or late-onset AD, will be assessed at baseline (t1), three months (t2), six months (t3) and nine months (t4) following treatment with TPS. For this monocenter, longitudinal study, participants will be recruited from an outpatient setting at a specialized psychiatric-psychotherapeutic clinic in Lower Saxony, Germany.

**Results:**

Preliminary data; repeated measures ANOVA; mean scores MoCA t1 = 13,9 vs. t2 = 14,1 vs. t3 = 14,5 vs. t4 = 14,4 (F(2, 66) = .749, n.s.), mean scores GDS t1 = 9,5 vs. t2 = 7,6 vs. t3 = 6,8 vs. t4 = 5,9 (F(2, 32 = 4.410, p ˂ .05). n = 40.

**Conclusions:**

The results suggest that cognitive performance can be stabilized and depressive symptoms can be reduced by using TPS in patients with mild-to-moderate Alzheimer’s disease. However, it is important to note that the results are based on a relatively small sample size and lack control conditions, which may limit the generalizability of the findings. Further research is needed to evaluate the effectiveness of TPS treatment for AD and to assess its potential as an add-on therapy that complements previous drug and non-drug approaches.

**Disclosure of Interest:**

V. Rößner-Ruff Employee of: No conflict value, C. Penkov Employee of: No conflict value, J. Michaelsen Employee of: No conflict value, J. Krieger Employee of: No conflict value, K. Friedrich Employee of: No conflict value, M. Ziegenbein Employee of: No conflict value

